# Authors’ Response to Peer Reviews of “Identification of COVID-19–Associated Hepatitis in Children as an Emerging Complication in the Wake of SARS-CoV-2 Infections: Ambispective Observational Study”

**DOI:** 10.2196/59327

**Published:** 2024-10-11

**Authors:** Sumit Kumar Rawat, Ajit Anand Asati, Nitu Mishra, Ashish Jain, Radha Kanta Ratho

**Affiliations:** 1 Bundelkhand Medical College and Hospital, Tilli Road, Sagar, 470002, India, 91 9303 212239; 2 Post Graduate Institute of Medical Education and Research, Chandigarh, India

**Keywords:** COVID-19, coronavirus, SARS-CoV-2, liver, hepatic, hepatitis, child, children, pediatric, pediatrics, retrospective, observational, jaundice, youth, inflammatory, inflammation

*This is the authors’ response to peer-review reports for “Identification of COVID-19–Associated Hepatitis in Children as an Emerging Complication in the Wake of SARS-CoV-2 Infections: Ambispective Observational Study.”*

## Round 1 Review

### Reviewer CG [1]

#### General Comments

*This paper [2] looks appropriate and relevant. Some corrections are needed. Additionally, none of the figures (except x-rays and study workflow) are properly visible on the PDF or Word version, so I cannot comment on their content. Please provide better-quality pictures, if possible.*

*Line numbers are not available, making the review and commenting much harder.*

#### Specific Comments

##### Major Comments

*1. Correction suggested: COVID-19 infection as a term does not exist; it is a disease and has been incorrectly used by the general public and the media. Instead, SARS-CoV-2 infection should be used as this is the correct scientific term. This correction could be performed throughout the entire article, but it was specifically first found in the following sentence: “During active COVID-19 infection, milder disease phenotype was observed without necessitating hospitalizations whereas more severe patterns were seen in cases with MIS-C often associated with prolonged need for hospital admissions.”*

Response: The manuscript has been thoroughly revised and rewritten, with the title changed to a shorter and more informative title. Correction of terminology: We appreciate your clarification regarding the terminology. We have replaced instances of “COVID-19 infection” with “SARS-CoV-2 infection” throughout the manuscript to adhere to scientific accuracy.

*2. Correction required in the following paragraph in the Discussion section: “With the emergence of newer VOC’S causing recurring waves of the pandemic, varied symptoms and post COVID-19 complications have been observed posing safety concerns even for the pediatric age group. In this scenario our study identified 37 cases with a unique presentation of acute hepatitis designated as CAH-C, whereas MIS-C could account for hepatitis in 10 cases amongst 15873 children screened in the district during the study period.” The number 15,873 in the Results section was previously mentioned as all screened patients regardless of age, and only 475 (2.99%) were actually children. Please correct or elaborate.*

Response: Thank you for pointing out this discrepancy. We have corrected the statement to accurately reflect that 5539 children were screened, out of which 475 were positive for SARS-CoV-2. We have also provided a clearer explanation in the *Discussion* section regarding the age breakdown of the screened population.

*3. Is it not clear if informed consent was required or acquired for the follow-up phase?*

Response: We apologize for the oversight. Informed consent was obtained from all participants or their guardians for the follow-up phase, and we have explicitly mentioned this in the manuscript.

*4. A few more details about the adverse outcomes of the 3 children with multiple inflammatory syndrome in children (MIS-C) would be appreciated by the reader. Were there any relevant circumstances or peculiarities for these cases?*

Response: We agree that providing more details on the adverse outcomes of the MIS-C cases would enhance the comprehensibility of our findings. We have included additional information about any relevant circumstances or peculiarities associated with these cases in the *Results* section.

##### Minor Comments

*5. References 7 and 8 might be wrong, as they do not relate to infections in children, as mentioned in the article. More appropriate references could be sought.*

Response: If necessary, we have substituted one of them with more relevant references, which were needed to indicate the magnitude of infections that occurred during the period.

*6. Sentence related to reference 17: Please correct to “at least 169” instead of “more than” to be exact with the terminology used in the original article. Otherwise, the meaning changes slightly.*

Response: Thank you for bringing this to our attention. We have ensured that the reference is correctly cited as 169 to maintain accuracy in the terminology.

*7. Reference 18 does not exist, or the link no longer works, at least from my side.*

Response: We have verified the availability of reference 18 and updated it accordingly if necessary.

*8. Correction suggested: “On laboratory investigation: 35/37 CAH-C cases had RTPCR test negative for SARS- CoV-2 by the time when admitted.” “When admitted” should be changed to “the time of admission.”*

Response: We acknowledge the suggested correction and have revised the sentence to read “35 out of 37 CAH-C cases had RTPCR tests negative for SARS-CoV-2 at the time of admission.”

*9. A reference is missing for this sentence in the Discussion section: “The other concern is the association of biological false positivity (BFP) which might pose a diagnostic dilemma in cases of other infectious diseases, wherein similar febrile illnesses including dengue, chikungunya and enteric fever remain endemic in developing countries. Both these facts warrant further elaborate studies amongst larger populations and also during the vaccine trails among children.”*

Response: We have added a reference to support the statement regarding the association of biological false positivity and its implications in diagnostic dilemmas.

### Reviewer CU [3]

*COVID-19–associated hepatitis in children (CAH-C) during the second wave of SARS-CoV-2 infections in Central India: is it a complication or transient phenomenon?*

*The topics of this paper are interesting but the results are not clear and robust. The structure and content must be revised, and the results have to be better explained by the authors before being reconsidered for publication.*

Response: We appreciate the thorough feedback provided. We have addressed each of the concerns raised and made necessary revisions to improve the clarity and coherence of the manuscript.

#### Title and Abstract

*The title has to be shorter.*

*The abstract has to clarify the goal, empirical results, and health and social policy for children to deal with this disease.*

Response: We have revised the title to be more concise and ensure that the abstract clearly outlines the study’s objectives, empirical results, and implications for health and social policy concerning children’s response to COVID-19 and associated hepatic diseases.

#### Introduction

*The introduction has to better clarify the research questions of this study and provide more theoretical background. The authors have to better describe the different sources of transmission dynamics of COVID-19 (eg, climate, air pollution, etc) and risk factors in society, which can accelerate the diffusion of this novel coronavirus in the environment and the emergence of this hepatic disease in children. After that, they can focus on the topics of this study to provide a correct analysis for fruitful discussion (see suggested readings that must be all read and used in the text).*

Response: We have tried to enhance the clarity of the research questions and provide additional theoretical background, particularly regarding the transmission dynamics of COVID-19 and the risk factors contributing to the emergence of hepatic diseases in children.

#### Methods

*The methods of this study are not clear. The Materials and Methods section must be restructured with following 3 sections only and in the same order:*

- *Sample and data*
- *Measures of variables*
- *Data analysis procedure*

*Inclusion and exclusion criteria can be better clarified for readers in a table.*

*The authors must avoid a lot of subheadings that create fragmentation and confusion. If necessary, they can use bullet points (same comment for the Results section and all sections).*

Response: We have restructured the *Materials and Methods* section as suggested, providing a clear delineation of sample and data, measures of variables, and data analysis procedures.

#### Results

*Titles of tables must always indicate the period under study to be clear.*

*For Table 1, insert a note to clarify the acronyms. The results are not clear, because they can be stronger with a comparative analysis.*

*…*

*The emergence of hepatitis in children can be investigated while considering the interaction of COVID-19 with air pollution in the environment and other factors. These aspects have to be discussed, analyzing hepatitis in a spatial dimension and considering pollution.*

Response: We have ensured that the titles of tables indicate the period under study, and we have inserted notes to clarify any acronyms used. We have also strengthened the comparative analysis in the *Results* section for a better interpretation of the findings.

#### Discussion

*First, the authors have to synthesize the main results in a simple table to ensure clarity for readers and then show what this study adds compared to other studies.*

Response: We have synthesized the main results in a simple table format and highlighted the contributions of our study compared to existing literature. Furthermore, we have incorporated spatial dimensions, such as air pollution, into the discussion of hepatitis emergence in children.

*The conclusion has to be an autonomous section. The conclusion does not have to be a summary, but the authors have to focus on manifold limitations of this study and provide suggestions of health, crisis management, and social policy to cope with COVID-19 and hepatitis in children.*

*Overall, then, the paper is interesting, but the structure is confusing, and the results are not strong without a comparative analysis. The theoretical framework is weak, and some results create confusion…the structure of the paper has to be improved. The study design, discussion, and presentation of results have to be clarified using the suggested comments.*

*I strongly suggest improving the paper by using all the comments (suggested papers have been included for the authors to read, and all should be used), which I will verify in depth, and then maybe it can be considered. If the paper is not improved as suggested, it will be dismissed.*

*Suggested readings of relevant papers that have to be read and all inserted in the text and references to improve, extend, and enrich the theory and discussion of results for implications of pandemic and postpandemic crisis management:*

- *Peters AL, Kim S, Mourya R, et al. Recent increase in incidence of severe acute hepatitis of unknown etiology in children is associated with infection with adenovirus and other nonhepatotropic viruses. J Pediatr. Aug 2023;259:113439. [doi: 10.1016/j.jpeds.2023.113439] [Medline: 37088181]*
- *Zeng G, Huang J. The recent outbreak of acute severe hepatitis in children of unknown origin. J Hepatol. Oct 2022;77(4):1213-121. [doi: 10.1016/j.jhep.2022.05.017] [Medline: 35644435]*
- *Coccia M. Sources, diffusion and prediction in COVID-19 pandemic: lessons learned to face next health emergency. AIMS Public Health. Mar 2, 2023;10(1):145-168. [doi: 10.3934/publichealth.2023012] [Medline: 37063362]*
- *Brüssow H. Non-A to E hepatitis in children: detecting a novel viral epidemic during the COVID-19 pandemic. Microb Biotechnol. Oct 2023;16(10):1879-1887. [doi: 10.1111/1751-7915.14329] [Medline: 37602673]*
- *Núñez-Delgado A, Bontempi E, Coccia M, Kumar M, Farkas K, Domingo JL. SARS-CoV-2 and other pathogenic microorganisms in the environment. Environ Res. Oct 2021;201:111606. [doi: 10.1016/j.envres.2021.111606] [Medline: 34181924]*
- *Gates S, Andreani J, Dewar R, et al. Postpandemic rebound of adeno-associated virus type 2 (AAV2) infections temporally associated with an outbreak of unexplained severe acute hepatitis in children in the United Kingdom. J Med Virol. Jul 2023;95(7):e28921. [doi: 10.1002/jmv.28921] [Medline: 37403889]*
- *Coccia M. Factors determining the diffusion of COVID-19 and suggested strategy to prevent future accelerated viral infectivity similar to COVID. Sci Total Environ. Aug 10, 2020;729:138474. [doi: 10.1016/j.scitotenv.2020.138474] [Medline: 32498152]*
- *Leiskau C, Tsaka S, Meyer-Ruhnke L, et al. Acute severe non-A-E-hepatitis of unknown origin in children – a 30-year retrospective observational study from north-west Germany. J Hepatol. May 2023;78(5):971-978. [doi: 10.1016/j.jhep.2022.12.012] [Medline: 36572350]*
- *Coccia M. How do low wind speeds and high levels of air pollution support the spread of COVID-19? Atmos Pollut Res. Jan 2021;12(1):437-445. [doi: 10.1016/j.apr.2020.10.002] [Medline: 33046960]*
- *Cooper S, Waisbourd-Zinman O. Reply to: severe hepatitis in children likely caused by HAdV-41 following SARS-CoV-2. J Pediatr Gastroenterol Nutr. Mar 1, 2023;76(3):e70. [doi: 10.1097/MPG.0000000000003689] [Medline: 36574220]*
- *Coccia M. Pandemic prevention: lessons from COVID-19. Encyclopedia. May 31, 2021;1(2):433-444. [doi: 10.3390/encyclopedia1020036]*
- *Mohammed FS, Karnsakul W, Mohammed S, Russo MW. Severe hepatitis in children likely caused by HAdV-41 following SARS-CoV-2 induced mitochondrial permeability transition. J Pediatr Gastroenterol Nutr. Mar 1, 2024;76(3):e69. [doi: 10.1097/MPG.0000000000003688] [Medline: 36574266]*

Response: The conclusion section has been revised to focus on the limitations of the study and provide recommendations for health, crisis management, and social policy in addressing COVID-19 and hepatic diseases in children.

## Round 2 Review

### Reviewer CG

*No further comments. The authors have considered my suggestions. I recommend the article for publication.*

Response: We thank both reviewers for their valuable feedback, which has significantly improved the quality and clarity of our manuscript.

A track version has not been uploaded since the manuscript has been thoroughly raised and entire sections have been rewritten.

## Abbreviations

CAH-C: COVID-19–associated hepatitis in children
MIS-C: multiple inflammatory syndrome in children
